# The combined molecular adjuvant CASAC enhances the CD8+ T cell response to a tumor-associated self-antigen in aged, immunosenescent mice

**DOI:** 10.1186/s12979-015-0033-0

**Published:** 2015-06-25

**Authors:** Gee Jun Tye, Kyriaki Ioannou, Eunice Amofah, Ruby Quartey-Papafio, Samantha J. Westrop, Pramila Krishnamurthy, Alistair Noble, Phillip M. Harrison, Karin M.L. Gaensler, Linda D. Barber, Farzin Farzaneh

**Affiliations:** Department of Haematological Medicine, King’s College London, Rayne Institute, London, UK; Institute for Research in Molecular Medicine, Universiti Sains Malaysia, George town, Malaysia; Medical Research Council and Asthma UK Centre in Allergic Mechanisms of Asthma, King’s College London, Guy’s Hospital, London, UK; Division of Transplant and Mucosal Cell Biology, King’s College London, London, UK; Department of Medicine, University of California, San Francisco School of Medicine, San Francisco, USA

**Keywords:** Immunotherapy, Vaccine, adjuvant, TLR, Immunosenescence, Ageing

## Abstract

**Background:**

Ineffective induction of T cell mediated immunity in older individuals remains a persistent challenge for vaccine development. Thus, there is a need for more efficient and sophisticated adjuvants that will complement novel vaccine strategies for the elderly. To this end, we have investigated a previously optimized, combined molecular adjuvant, CASAC (Combined Adjuvant for Synergistic Activation of Cellular immunity), incorporating two complementary Toll-like receptor agonists, CpG and polyI:C, a class-II epitope, and interferon (IFN)-γ in aged mice.

**Findings:**

In aged mice with typical features of immunosenescence, antigen specific CD8+ T cell responses were stimulated after serial vaccinations with CASAC or Complete/Incomplete Freund’s Adjuvant (CFA/IFA) and a class I epitope, deriving either from ovalbumin (SIINFEKL, SIL) or the melanoma-associated self-antigen, tyrosinase-related protein-2 (SVYDFFVWL, SVL). Pentamer analysis revealed that aged, CASAC/SIL-vaccinated animals had substantially higher frequencies of H-2K^b^/SIL-specific CD8+ T cells compared to the CFA/IFA-vaccinated groups. Similarly, higher frequencies of H-2K^b^/SVL-pentamer+ and IFN-γ+ CD8+ T cells were detected in the aged, CASAC + SVL-vaccinated mice than in their CFA/IFA-vaccinated counterparts. In both antigen settings, CASAC promoted significantly better functional CD8+ T cell activity.

**Conclusion:**

These studies demonstrate that functional CD8+ T cells, specific for both foreign and tumour-associated self-antigens, can be effectively induced in aged immunosenescent mice using the novel multi-factorial adjuvant CASAC.

## Findings

### Introduction

The sequelae of immunosenescence in older individuals, including increased morbidity and mortality from infection and malignancy, represent a critically important public health problem [1]. As a consequence of accumulated dysfunctions in the immune system during ageing, vaccination of older individuals is less efficacious than in the young [2, 3]. Previous attempts to increase vaccination efficacy by incorporating various adjuvants, including alum, have shown only modest success [2]. Thus, there is an unmet need for new vaccine strategies for the elderly.

Immunosenescence is characterised by decline in both adaptive and innate immune functions [4, 5]. Innate immune responses are activated, mainly, by stimulation of Toll-like receptors (TLRs) [6], the expression and function of which declines with age [7]. Dendritic cells (DCs) from both young and aged individuals exhibit comparable activation in response to most TLR ligands, and are equally capable of direct and cross-presentation of antigens to T cells *in vitro* [8], underscoring the likely importance of TLR-induced DC activation in promoting adaptive immunity. TLR stimulation is therefore a promising strategy to enhance vaccine efficacy in the elderly. Combinations of TLR agonists may be especially effective, as demonstrated in animal models and clinical trials [6, 9–13].

We previously showed that triggering of multiple TLRs, using a combined adjuvant for synergistic activation of cellular immunity (CASAC), incorporating CpG, polyI:C, interferon (IFN)-γ and MHC-class I and II peptides, results in potent cytotoxic T cell-mediated immunity in young mice [14]. Optimization of the adjuvant formulation and investigation of mechanism of action were also performed [14]. We now report the ability of CASAC to improve vaccination-induced responses in aged mice by promoting induction of antigen-specific cellular immunity to both foreign and self tumour-associated peptide antigens.

## Methods

### Animals and vaccination procedures

Young (6–8 weeks old) and aged (18–22 months old) wild-type C57BL/6 female mice were purchased from Harlan, UK. All animal procedures were performed according to UK Home Office and institutional regulations.

CASAC vaccine comprised of an oil-in-water emulsion consisting of Tween-80 and squalene (all Sigma, UK), as previously described [14]. The tween/squalene mixture was sonicated and mixed at a 1:1 ratio with PBS containing: 50 μg polyI:C (TLR3 agonist; Sigma), 25 μg CpG 1826 (TLR9 agonist; Eurofins, UK), 100 ng mouse recombinant IFN-γ (Peprotech, UK), 100 μg ISQAVHAAHAEINEAGR (ovalbumin (OVA)-derived MHC-class II (H-2IA^b^)-restricted peptide) and 100 μg SIINFEKL (SIL; OVA-derived MHC-class I (H-2K^b^)-restricted peptide) or SVYDFFVWL (SVL; tyrosinase related protein (TRP)-2-derived MHC-class I (H-2K^b^)-restricted peptide; all PPR, UK). Alternatively, 100 μg of SIL or SVL was emulsified with Complete Freund’s Adjuvant (CFA) for the first vaccination, and Incomplete FA (IFA; all Sigma) for subsequent vaccinations at a 1:1 (vol/vol) ratio. All vaccine formulations were administered intradermally on days 0, 10, 20 and 30 (±1 day) in 100 μL final volume (50 μL/flank).

### Flow cytometric analysis

Cell enumeration was performed in whole blood samples using Flow-Count™ beads (Beckman Coulter, UK) according to manufacturer’s instructions. After red blood cell lysis, mononuclear cells were stained with anti-CD3/eFluor 450, anti-CD4/FITC and anti-CD8a/PerCP-Cy5.5 monoclonal antibodies (mAb) (all eBioscience, USA). Expression of PD-1, KLRG1 and LAG-3 was assessed in whole blood samples after staining with anti-CD3/eFluor 450, anti-CD8a/PerCP-Cy5.5, anti-PD-1/FITC, KLRG-1/APC and anti-LAG-3/PE mAbs (all eBioscience). Pentamer analysis was performed as previously described [14], using H-2K^b^/SIINFEKL or H-2K^b^/SVYDFFVWL Pro5 pentamer/PE (ProImmune, UK).

To assess peptide-induced intracellular accumulation of IFN-γ by CD8+ T cells, splenocytes were stimulated with 1 μg/mL SVL peptide, 0.5 μg/mL co-stimulatory anti-CD28 antibody (eBioscience) in the presence of GolgiPlug (BD Biosciences, Belgium) for 5 h prior to fixation, permeabilization, and staining with anti-CD3/eFluor 450, anti-CD8a/PerCp-Cy5.5 and anti-IFN-γ/PE mAbs (eBioscience).

Samples were analysed using a FACSCantoII (BD Biosciences) and FACSDiva (BD Biosciences) or FlowJo (Treestar, OR) software.

### *In vivo* cytotoxicity assay

The *in vivo* cytotoxicity assay was performed as previously described [14].

### Statistical analysis

The Mann-Whitney *U* test (GraphPad Prism, USA) was used to compare distributions, with p < 0.05 considered significant.

## Results and discussion

**Fig. 1:**
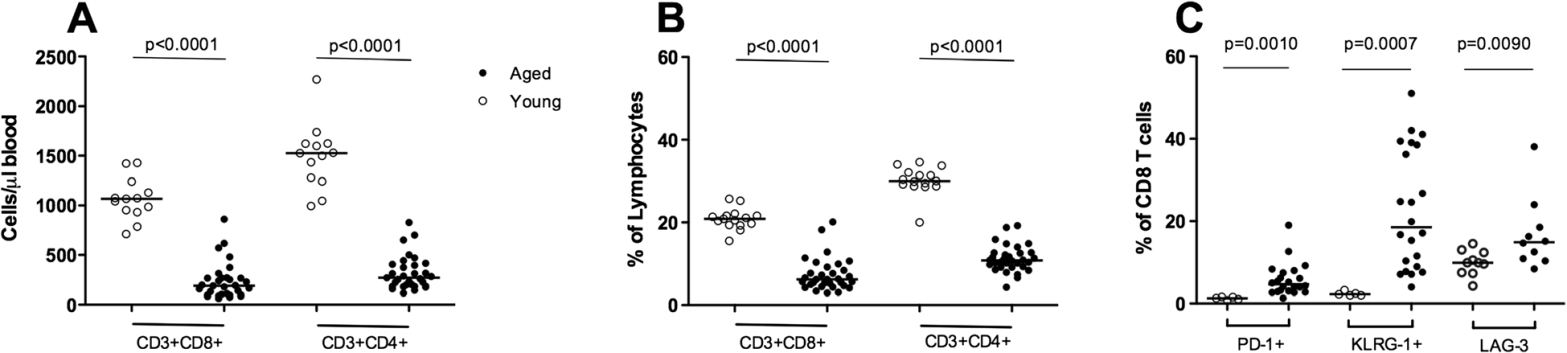
Age-associated differences in peripheral blood T cell subsets. The absolute numbers (**a**) and percentages (**b**) of CD4+ and CD8+ T cells were determined in blood samples from young (open circles) and aged (filled circles) C57BL/6 mice by flow cytometric analysis using counting beads. **c** Expression of PD-1, KLRG-1 and LAG-3 on CD8+ T cells in young and aged mice. Each symbol represents an individual mouse and the median is indicated by a horizontal line. Data is pooled from 2 independent experiments. The Mann-Whitney *U* test was used to compare distributions

Previous studies have shown that immunosenescence associated with increasing age is especially pronounced within the T cell compartment [15–17]. Consistent with these reports, aged C57BL/6 mice used in our study had significantly lower CD4+ (median 270 cells/μL blood) and CD8+ (median 189 cells/μL of blood) T cell numbers, compared to young mice (1527 CD4+/μL blood; *p* < 0.0001 and 1067 CD8+/μL blood; *p* < 0.0001) (Fig. 1a). CD4+ and CD8+ T cell percentages were also decreased significantly in aged (median 10.8 and 6.2 % of all lymphocytes, respectively), compared to young mice (30.00 %; *p* < 0.0001 and 20.90 %; *p* < 0.0001, respectively) (Fig. 1b). Expression of PD-1, KLRG-1 and LAG-3, indicative of T cell exhaustion and anergy [18, 19], was significantly elevated on CD8+ T cells in aged mice (Fig. 1c) compared to young counterparts.

### CASAC enhances responses to a foreign antigenic CD8+ T cell epitope in aged mice

**Fig. 2:**
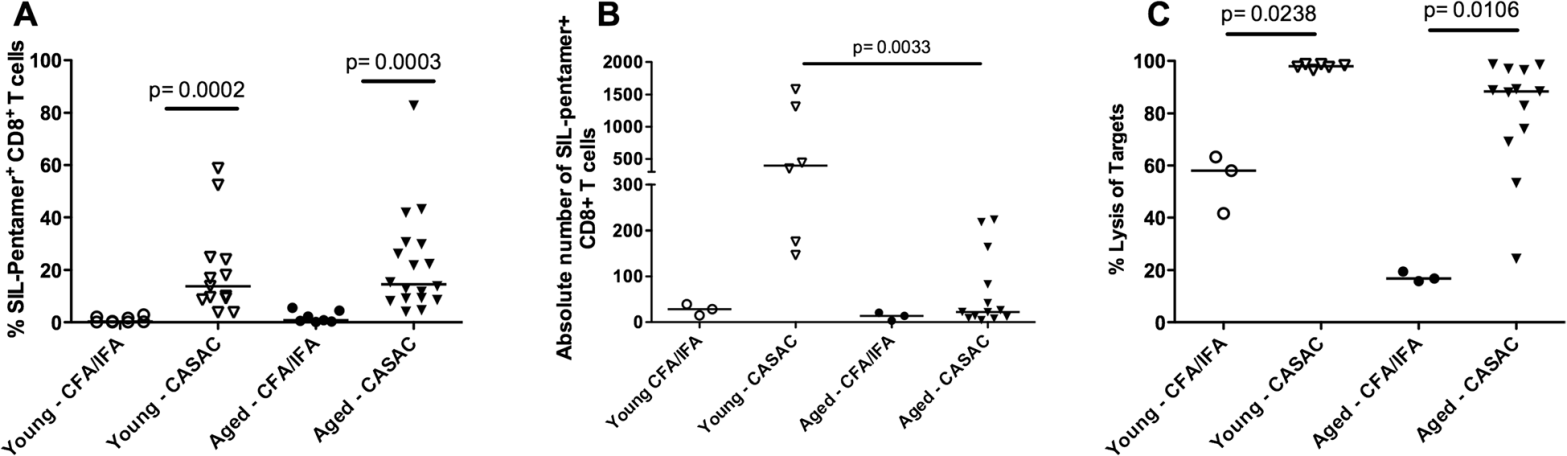
CASAC enhances CD8+ T cell responses to a foreign antigenic epitope in aged mice. Young (open symbols) and aged (filled symbols) C57BL/6 mice were vaccinated twice with the SIL peptide from OVA in combination with either CFA/IFA (circles) or CASAC (triangles). **a** The percentage of H-2K^b^/SIL-pentamer + CD8+ T cells was assessed after staining of peripheral blood samples with H-2K^b^/SIL-pentamer. **b** Absolute numbers of H-2K^b^/SIL-pentamer + CD8+ T cells were enumerated by flow cytometry. **c** % Lysis of target cells was assessed using CFSE^low^–SIL-loaded and CFSE^high^-SVL-loaded splenocytes, as antigen-specific and control targets, respectively. Both populations were mixed and injected iv into the immunised and control mice. Eighteen hours later, all spleens were harvested and splenocytes were analysed for their CFSE content by flow cytometry. The percent target lysis was calculated with the following formula: % lysis = 1 – [(number of CFSE^low^/ number of CSFE^high^ in immunised animal)/(number of CFSE^low^/ number of CSFE^high^ in unimmunised animal)] × 100. Each symbol represents an individual mouse and the median is indicated by a horizontal line. Data is pooled from 2 independent experiments. The Mann-Whitney *U* test was used to compare distributions

CASAC was previously shown to effectively promote T cell immunity to the foreign antigen OVA in young mice [14]. We therefore investigated whether CASAC augments responses to the immunogenic OVA peptide SIL [20] in aged mice, compared to CFA/IFA as a conventional adjuvant control [21]. Using our previously described vaccination protocol [14], young and aged C57BL/6 mice were vaccinated twice at a 10-day interval with SIL, combined either with CFA/IFA or CASAC. The distribution of percentages of H-2K^b^/SIL-pentamer+ CD8+ T cells was significantly higher (*p* = 0.0003) in the CASAC-vaccinated group (median 14.55 %), compared to CFA/IFA vaccinations (0.80 %). As shown previously [14], distribution of H-2K^b^/SIL-pentamer+ CD8+ T cell frequencies was also found to be significantly higher (*p* = 0.0002) in young mice vaccinated with CASAC (median 13.80 %), compared to their CFA/IFA-treated counterparts (0.29 %). Of importance, the H-2K^b^/SIL-pentamer+ CD8+ T cell frequencies induced by CASAC vaccinations in both young and aged mice were similar. Although similar percentages were induced in both CASAC-vaccinated cohorts, the absolute numbers of SIL-specific CD8+ T cells (Fig. 2b) showed a significantly lower (p = 0.0033) distribution in aged mice (median 22.30) compared to their young counterparts (396.3). This was due to the lower total numbers of CD8+ T cells in aged mice (Fig. 1a). The function of SIL-specific T cells was assessed by performing *in vivo* cytolytic assays. Aged mice vaccinated with CASAC showed higher distribution of antigen-specific cytolytic activity (median 88.5 %; Fig. 2c) compared to the CFA/IFA-vaccinated mice (16.8 %; *p* = 0.0106). Similar outcomes were observed in the young groups (98.0 and 58.1 % for CASAC and CFA/IFA vaccinated groups, respectively; *p* = 0.0238). These results show that CASAC effectively induced CD8+ T cell mediated immunity to a foreign antigen in both aged and young mice.

### CASAC enhances responses to a tumor-associated CD8+ T cell epitope in the aged mice

The need for efficient vaccine adjuvants applies not only to infectious disease but also to cancer, a disease with increasing age-associated incidence [22]. It is therefore imperative that new adjuvants incorporated in anti-cancer vaccines are assessed for efficacy in aged immunosenescent individuals.

**Fig. 3:**
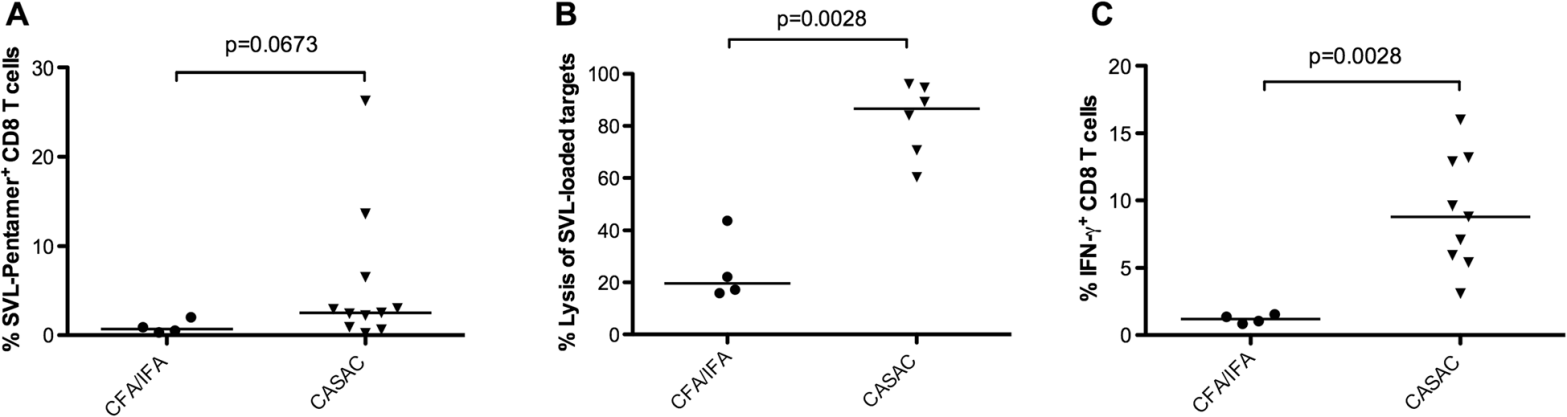
CASAC enhances CD8+ T cell responses to a tumour-associated epitope in aged mice. Aged C57BL/6 mice were vaccinated four times with the SVL peptide from TRP-2 in combination with either CFA/IFA (circles) or CASAC (triangles). **a** The percentage of H-2K^b^/SVL-pentamer + CD8+ T cells was assessed after staining with H-2K^b^/SVL-pentamer. **b** % Lysis of target cells was assessed (as explained in the legend to Fig. 2c) using CFSE^low^–SVL-loaded and CFSE^high^-SIL-loaded splenocytes as antigen-specific and control targets, respectively. **c** Production and intracellular accumulation of IFN-γ in CD8^+^ T cells in response to the vaccinated peptide was assessed using flow cytometry, after *in vitro* SVL peptide stimulation of splenocytes for 5 h. Each symbol represents an individual mouse and the median is indicated by a horizontal line. Data is pooled from 2 independent experiments. The Mann–Whitney *U* test was used to compare distributions

We have previously shown that immune tolerance to the melanoma-associated self-antigen TRP-2 can be overcome by vaccination of young mice with the TRP-2 peptide SVL combined with CASAC [14]. Functional SVL-specific CD8+ T cells were detected after two rounds of vaccinations; however, responses were of variable magnitude and required vaccination with the higher peptide dose of 400 μg. We therefore performed further optimization studies in young mice and found four rounds of vaccination, using 100 μg SVL peptide with CASAC, consistently induced higher frequencies of SVL-specific CD8+ T cells (data not shown). All studies with aged mice were performed with four rounds of vaccination using 100 μg SVL peptide in combination with either CASAC or CFA/IFA. Distribution of SVL-specific CD8+ T cell frequencies was higher in the SVL + CASAC-vaccinated group (median 2.5 %) compared to the SVL + CFA/IFA-vaccinated group (0.7 %), although the difference did not reach statistical significance (*p* = 0.0673; Fig. 3a). *In vivo* cytotoxicity studies (Fig. 3b) showed that lysis of SVL-loaded splenocytes was significantly higher in SVL + CASAC-vaccinated (median 86.6 %) compared to SVL + CFA/IFA-vaccinated aged mice (median 19.7 %; *p* = 0.0028). Additionally, distribution of IFN-γ+ CD8+ T cell frequencies after *in vitro* SVL peptide stimulation was significantly higher in the SVL + CASAC-vaccinated mice (median 8.9 %; Fig. 3c) compared to CFA/IFA-vaccinated mice (1.2 %; *p* = 0.0028). Thus, vaccination of aged mice with the tumour-associated SVL peptide and CASAC induced antigen-specific CD8+ T cells that had considerably better functional activity than CD8+ T cells induced by SVL + CFA/IFA-vaccination.

In conclusion, we have demonstrated that our combined molecular adjuvant CASAC effectively promotes functional antigen-specific CD8+ T cell responses to vaccination with peptides in aged mice, despite their immunosenescent phenotype. CASAC improved responses in aged mice not only to a highly immunogenic foreign antigen, but also to the tumour-associated self-antigen TRP-2 whose immunogenicity is being evaluated in clinical trials [23]. Restoration of response to vaccination in immunosenescent aged mice by CASAC likely reflects the benefits of multiple TLR triggering on DC function [14, 24, 25] and provision of IFN-γ could substitute for lack of IFN-γ from CD8+ memory cells during the early phase of immune response. Since CASAC comprises a combination of agents that individually are approved for human use, our findings suggest that a CASAC-based vaccination strategy may be amenable to rapid clinical translation, particularly against chronically experienced antigens such as persistent infections or tumour-associated antigens in older people.

## Abbreviations

CASAC: Combined Adjuvant for Synergistic Activation of Cellular Immunity
CFA: Complete Freund’s Adjuvant
DC: Dendritic Cell
IFA: Incomplete Freund’s Adjuvant
IFN: Interferon
mAb: Monoclonal Antibody
OVA: Ovalbumin
SIL: SIINFEKL
SVL: SVYDFFVWL
TLR: Toll-Like Receptor
TRP-2: Tyrosinase-Related Protein 2
